# Emerging Concepts in Therapeutic Interventions for Idiopathic Pulmonary Fibrosis

**DOI:** 10.1055/a-2651-1937

**Published:** 2025-08-12

**Authors:** Cody A. Schott, Michael P. Mohning, Joseph C. Cooley

**Affiliations:** 1 Division of Pulmonary Sciences and Critical Care Medicine, Department of Medicine, University of Colorado, Aurora, Colorado; 2 Division of Pulmonary, Critical Care, and Sleep Medicine, Department of Medicine, National Jewish Health, Denver, Colorado

**Keywords:** idiopathic pulmonary fibrosis, interstitial lung disease, anti-fibrotic, preclinical models, pulmonary fibrosis

## Abstract

Idiopathic pulmonary fibrosis (IPF) is a rare but devastating diagnosis for patients with only two approved drug therapies. Extensive preclinical studies have identified and characterized novel pathways driving IPF pathogenesis, and researchers have identified several new promising therapeutic targets to help treat IPF. However, translating these preclinical models into viable treatment modalities has proven challenging. This review will address the evolving nature of IPF research, examine the preclinical studies and their target pathways that have advanced to clinical trials, and address the translational gap that has limited the success of novel therapeutic trials for IPF.

Idiopathic pulmonary fibrosis (IPF) is the most common etiology of fibrotic lung disease worldwide. Incidence is 1 to 13 cases per 100,000, with prevalence ranging from 3 to 45 cases per 100,000. 1 Risk factors for disease development include age, male sex, inhalational exposures (including tobacco), comorbidities, and genetic factors. 2 Survival is often quoted as 3 to 5 years; however, this is likely an underestimate due to earlier identification, effective drug therapies, effective lifestyle modifications, including pulmonary rehab, and treatment of comorbidities. 3 4 5 6 7 8 9 While there are only two FDA-approved therapies for IPF, comprehensive preclinical studies have identified exciting and novel therapeutic targets. As a result, several new promising drugs have advanced to clinical trials, which we will summarize in this review ( Fig. 1 ; Table 1 ).

**Fig. 1:**
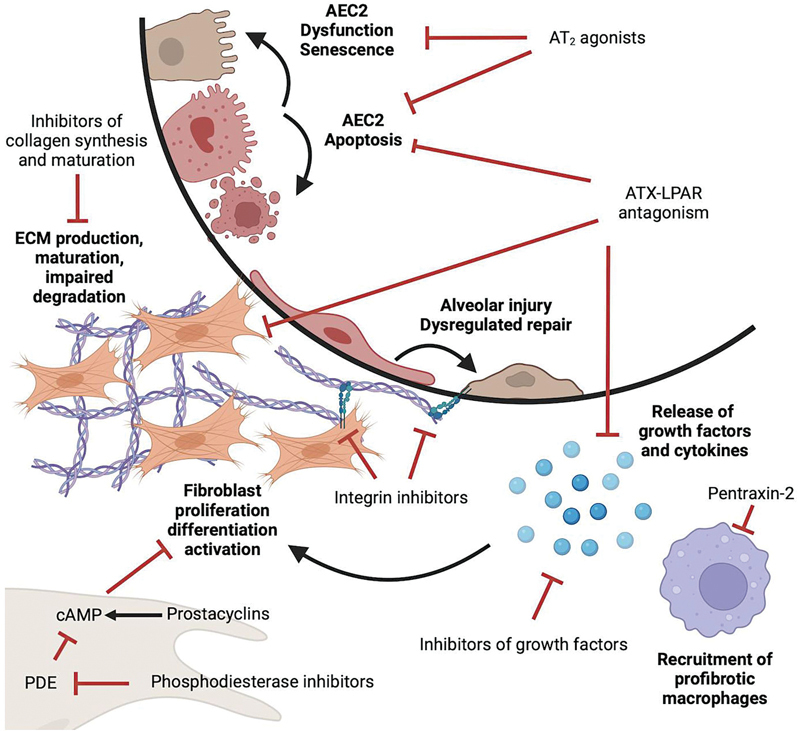
Schematic of critical events in IPF pathogenesis and how therapeutic categories target them. Repetitive epithelial injury leads to maladaptive healing, AEC2 dysfunction, senescence, and apoptosis. Pro-inflammatory cell types, including macrophages, epithelial cells, and fibroblasts, produce profibrotic cytokines which promote fibroblast proliferation, differentiation into myofibroblasts, and activation. Fibroblasts produce excessive ECM, resulting in an imbalance between production and degradation. AEC2, type 2 alveolar epithelial cells; AT2, angiotensin 2 receptor; ATX, autotaxin; cAMP, cyclic adenosine monophosphate; ECM, extracellular matrix; LPAR, lysophosphatidic acid receptor; PDE, phosphodiesterase. (Created in BioRender by Cooley J. [2025] https://BioRender.com/5me0sry .)

**Table 1:** Select phases II and III trials evaluating the efficacy of targeted therapies in IPF

Drug	Other names	Mechanism of action	Clinical trial	Phase	Status a	Primary endpoint	Duration	Primary met?	Background therapy	Notes
Pirfenidone 58 59	Esbriet, Pirespa, Etuary	Prevents TGF-β signaling	CAPACITY (1 and 2)	III	FDA approved	Change in predicted FVC	72 wk	Yes	None	CAPACITY 1 only showed benefit at earlier timepoints
			ASCEND			Change in FVC or death	52 wk	Yes	None	Improved progression-free survival and 6MWT decline with pirfenidone treatment
Nintedanib 73	BIBF 1120, Ofev, Vargatef	Inhibits PDGF, VEGF, and FGF receptors	INPULSIS (1 and 2)	III	FDA approved	Rate of decline in FVC	52 wk	Yes	None	
Saracatinib	AZD0530	Inhibits the Src family of tyrosine kinases	STOP-IPF	Ib/IIa	Active, not recruiting	Change in FVC from baseline	24 wk	Pending	None	
Pamrevlumab 86	FG-3019	Monoclonal antibody against CTGF	ZEPHYRUS (1 and 2)	III	Terminated	Change in FVC from baseline	48 wk	No	None; allowed after study initiation for worsening respiratory status	Only ZEPHYRUS-1 results available
Vixarelimab	RG6536	Monoclonal antibody against OSM receptor	NCT05785624	II	Recruiting	Change in FVC from baseline	52 wk	Pending	Stable background therapy allowed	
Treprostinil	Tyvaso	Prostacyclin analogue	TETON (1 and 2)	III	Active, not recruiting	Change in FVC from baseline	52 wk	Pending	Stable background therapy allowed	
Nerandomilast 114	BI1015550	PDE4B inhibitor	FIBRONEER-IPF	III	Completed	Change in FVC from baseline	52 wk	Yes	Stable background therapy allowed	Data yet to be formally published
Sildenafil 118 119	Revatio, viagra, others	PDE5 inhibitor	STEP-IPF	III	Completed	Increase in 6MWT by 20% or more	12 wk	No	Stable background therapy allowed	Improved arterial oxygen saturation, DL _CO_ , and degree of dyspnea
			INSTAGE-IPF	III	Completed	Change in SGRQ score from baseline	12 wk	No	Combination therapy with nintedanib	DL _CO_ < 35% predicted inclusion criteria
Ifetroban		Thromboxane receptor antagonist	NCT05571059	II	Recruiting	Change in FVC from baseline	52 wk	Pending	Stable background therapy allowed	Patients on anticoagulation and antiplatelet therapy were excluded
Bosentan 132 133	Tracleer, stayveer, safebo	Endothelin receptor antagonist	BUILD-1	III	Completed	Change in exercise capacity from baseline	12 mo	No	None	Improved time to death or disease progression in those diagnosed by surgical lung biopsy
			BUILD-3	III	Completed	Time to IPF worsening	Variable	No	None	Patients diagnosed by surgical lung biopsy
Ambrisentan 134	Letairis, volibris, pulmonext	Endothelin receptor antagonist	ARTEMIS-IPF	III	Terminated	Time to disease progression	Variable	No	None	Interim analysis with increased risk of disease progression in the ambrisentan group
Macitentan 135	Opsumit	Endothelin receptor antagonist	MUSIC	II	Completed	Change in FVC from baseline	12 mo	No	None	Patients diagnosed by surgical lung biopsy
Ziritaxestat 153	GLPG1690	Autotaxin inhibitor	ISABELA (1 and 2)	III	Terminated	Rate of decline in FVC	52 wk	No	Stable therapy allowed	Terminated early for lack of efficacy and increased mortality in ziritaxestat groups
Fipaxalparant	HZN-825	LPAR1 antagonist	NCT05032066	II	Terminated	Change in predicted FVC	52 wk	No	Stable therapy allowed	Terminated early, no results released to date
Admilparant 156	BMS986-278	LPAR1 antagonist	NCT04308681	II	Completed	Change in predicted FVC	26 wk	Yes	Stable therapy allowed	30 and 60 mg doses tested
BG00011 167		Monoclonal antibody against integrin αvβ6	NCT03573505	IIb	Terminated	Change in FVC from baseline	52 wk	No	Stable therapy allowed	Terminated early; signal for worsening at 26-wk analysis
Bexotegrast	PLN-74809	Inhibitor of integrins αvβ6 and αvβ1	BEACON-IPF	II	Active, not recruiting	Change in FVC from baseline	52 wk	Pending	Stable therapy allowed	Phase IIa INTEGRIS-IPF demonstrated safety and tolerability
DWN12088		Prolyl-tRNA synthetase inhibitor, disrupting collagen translation	NCT05389215	II	Recruiting	Rate of decline in FVC	24 wk	Pending	Stable therapy allowed	
Simtuzumab 174	GS 6624	LOXL2 inhibitor, disrupting collagen crosslinking	RAINIER	II	Terminated	Progression-free survival	Variable	No	Stable therapy allowed	Terminated early for lack of efficacy
GSK3915393		Transglutaminase-2 inhibitor,	TRANSFORM	II	Recruiting	Change in FVC from baseline	26 wk	Pending	Stable therapy allowed	
Setanaxib	GKT137831	Nox1 and Nox4 inhibitor	NCT03865927	II	Completed	Change in the concentration of the marker of oxidative stress	24 wk	Pending	Not specified	
Buloxibutid	C21	Angiotensin type 2 receptor agonist	ASPIRE-IPF	II	Recruiting	Change in FVC from baseline	52 wk	Pending	Nintedanib allowed	Pirfenidone use is excluded due to drug-drug interactions
Zinpentraxin alfa 238	rhPTX-2, PRM-151	Recombinant human pentraxin-2	STARSCAPE	III	Terminated	Change in FVC from baseline	52 wk	No	Stable therapy allowed	Terminated early for lack of efficacy

Abbreviations: 6MWT, 6-minute walk test distance; DL _CO_ , diffusing capacity of the lungs for carbon monoxide; FVC, forced vital capacity; SGRQ, Saint George Respiratory Questionnaire.

*Source* : Trial parameters from clinicaltrials.gov.

a At the time of manuscript submission.

Clinically, IPF is defined radiographically and histologically by the presence of usual interstitial pneumonia without evidence of underlying exposure or rheumatologic cause. 10 These findings manifest as progressive reticular changes in the lung, traction bronchiectasis and bronchiolectasis, and ultimately, honeycomb cyst formation. 10 11 Symptoms manifest as dry cough, dyspnea, and hypoxia. Physiology most frequently demonstrates pulmonary restriction and gas exchange abnormalities with reduced total lung capacity, forced vital capacity (FVC), and low diffusing capacity of the lung for carbon monoxide (DL _CO_ ). 12 Diagnosis is made using careful appraisal of the clinical history, physical exam findings, pulmonary function tests, imaging, and relevant serologic data. When considering other etiologies, surgical lung biopsy may be performed for definitive diagnosis. In a small cohort of patients who received clinical and histopathologic diagnoses from biopsy, a clinical assessment alone was accurate in 62% of patients diagnosed with IPF. 13 Critically, the symptoms and physiologic changes associated with IPF frequently occur prior to formal diagnosis. 14 Clinical progression is tracked by trending patient-reported outcomes, spirometry, DL _CO_ , 6-minute walk test distance (6MWT), and radiographic evidence of fibrosis, which form the basis for clinical trial outcomes in IPF research. 15

The pathogenesis of IPF has not been fully elucidated, but the working hypothesis is that a genetically susceptible individual undergoes repetitive epithelial injury, resulting in pro-fibrotic signaling, accumulation of pro-fibrotic fibroblasts, and an imbalance between collagen synthesis and degradation. 2 16 17 Preclinical studies utilize in vitro, ex vivo, and in vivo approaches to identify mechanisms driving fibrotic change. 18 19 20 21 In vivo mouse models have been foundational in identifying pathophysiologic mechanisms of fibrosis. In vivo models of pulmonary fibrosis utilize injurious exposures including chemical (bleomycin), radiation, inhalation of damaging substances (silica and asbestos), or genetic modalities to induce fibrosis. 20 21 The most well-characterized of these models utilizes a single dose of intratracheal bleomycin, a chemotherapeutic agent causing double-stranded DNA breaks. 22 23 24 25 26 Bleomycin-induced lung injury can be broken into four overlapping phases. After an acute inflammatory phase (days: 0–7), a fibroproliferative phase ensues (days: 3–14), followed by an established fibrotic phase (days: 14–28) which resolves over time (days: 42–56), leaving mice with near normal lung architecture. 21 24 26 It shares several phenotypic similarities with IPF during the fibrotic phase, including epithelial cell damage and remodeling, pro-fibrotic signaling, fibroblast proliferation, accumulation of extracellular matrix (ECM), and architectural distortion. 23 When interpreting in vivo studies using bleomycin in mice, the timing of intervention is important. We will use the following nomenclature when describing in vivo mouse studies with bleomycin, consistent with Kolb et al. 23 Inhibiting development of fibrosis will refer to interventions made prior to bleomycin and up to 6 days postbleomycin. Therapeutically reducing fibrosis refers to interventions initiated 7 days or more postbleomycin. However, if an intervention is initiated after 7 days postbleomycin and the analysis occurs at least 6 weeks postbleomycin, we will refer to this as hastening resolution.

Pirfenidone and nintedanib remain the only two FDA-approved therapies for the treatment of IPF. They both slow fibrosis progression and likely have a mortality benefit in IPF, but don't reverse established progressive fibrosis. 8 27 28 29 30 31 As of March 2025, review of clinicaltrials.gov demonstrates 9 active and 20 completed phase III clinical trials. This review will explore promising therapeutic avenues, summarizing the (1) targeted mechanisms, (2) preclinical data, and (3) clinical trial results. Exploring the path from bench to bedside will highlight the diverse pathways driving pulmonary fibrosis in preclinical models and identify promising clinical trials.

## Growth Factors and Cytokines

Several pro-fibrotic signaling pathways that contribute to epithelial injury, fibroblast recruitment, proliferation, and resilience have been implicated in the pathogenesis of pulmonary fibrosis. Signaling pathways have formed the backbone of pulmonary fibrosis research since the identification of TGF-β as a key mediator of fibrosis. 32 33 Increased levels of TGF-β have been identified in IPF lungs, and overexpression of TGF-β in animal models (both genetic and through adenoviral delivery) results in the development of fibrosis. 34 35 36 37 38 TGF-β is produced by several cell types integral to the pathogenesis of IPF, namely damaged alveolar epithelial cells, pro-fibrotic macrophages, and fibroblasts. 38 39 Upon binding to its receptor, TGF-β activates Smad2 and Smad3 via phosphorylation, allowing for translocation to the nucleus and promoting transcription of several fibrosis-related genes, including collagen synthesis. 40 41 42 43 Loss of Smad3 is protective against the development of bleomycin fibrosis in mice. 44 TGF-β also drives epithelial-to-mesenchymal transition, fibroblast proliferation, differentiation into myofibroblasts, and fibroblast activation. 45 46 47 48 While TGF-β has long been considered a target for IPF, there have been limited clinical studies to date directly targeting TGF-β, largely due to concerns about on target effects outside of lung fibrosis. 49 TRK-250, an inhaled small-interfering RNA (siRNA) molecule blocking TGF-β production, completed phase I trials but has not advanced further. 50

While the precise mechanism of action has not been fully elucidated, pirfenidone has been shown to decrease expression of TGF-β in vitro and in vivo models of fibrosis, thereby decreasing production of ECM components like collagen, fibronectin, and tenascin-C. 51 52 53 54 Additionally, pirfenidone decreases expression of pro-fibrotic growth factors like platelet-derived growth factor (PDGF) and fibroblast growth factor 2. 53 55 56 Pirfenidone has also been shown to decrease the expression of some matrix metalloproteinases (MMP), reduce inflammatory cells and cytokine expression, which have been excellently reviewed elsewhere. 57 Efficacy of pirfenidone in IPF was evaluated in three phase III randomized controlled trials (RCTs), ASCEND and two CAPACITY RCTs. 58 59 Pooled results demonstrate that at 1 year, placebo-treated patients experienced an FVC decline of 363 mL, pirfenidone-treated patients experienced a decline of 216 mL, and no decline was observed in 59.3% more pirfenidone-treated patients. 60 Treatment was well tolerated, and the most common side effects were gastrointestinal-related and rash. 60 Nebulized pirfenidone is currently being studied as an alternative to the oral agent to reduce side effects, predominantly upper GI distress. 61 A deuterated formulation, deupirfenidone, is also being studied for improved pharmacokinetic and safety profiles in the ELEVATE-IPF trial, with promising early results noted in a press release. 62 63

Nintedanib is a tyrosine kinase inhibitor that targets several additional growth factors implicated in IPF, namely PDGF, FGF, and vascular endothelial growth factor (VEGF). PDGF RNA expression is increased in the lungs of IPF patients and is derived from alveolar macrophages and epithelial cells. 64 In response to PDGF stimulation, fibroblasts proliferate, produce collagens, and a-SMA. 64 65 66 The FGF family of growth factors has multiple effects on fibroblasts, including fibroblast proliferation and collagen production. 47 67 FGF-9 and FGF-18 are also known to promote fibroblast survival. 68 VEGF is produced by type II alveolar epithelial cells and fibroblasts in the lung, but its effects on fibrosis are unclear, as some investigators report pro-fibrotic effects and others anti-fibrotic effects. 69 70 By antagonizing the receptors of PDGF, FGF, and VEGF, nintedanib exerts anti-fibrotic effects. After bleomycin administration in mice, nintedanib inhibited the development of fibrosis and therapeutically reduced fibrosis in a dose-dependent manner. 71 72 Nintedanib treatment for IPF was evaluated in two phase III RCTs, INPULSIS-1 and 2, which demonstrated that nintedanib reduced the rate of FVC decline in IPF patients (−240 mL/year in placebo group vs. −115 mL/year in nintedanib group). 73 Its primary side effect was diarrhea (19% in the placebo group and 62% in the nintedanib group), which significantly restricts its use clinically.

The Src-family tyrosine kinases modulate several pro-fibrotic signaling pathways, including PDGF, VEGF, FGF, and epidermal growth factor. 74 75 76 77 78 Saracatanib, a c-Src inhibitor, therapeutically reduced fibrosis after both bleomycin and recombinant-TGF-β adenovirus induced fibrosis in mice. 79 In these animal models, saracatinib demonstrated equal to improved efficacy compared to nintedanib and pirfenidone in therapeutically reducing fibrosis. 79 A phase Ib/IIa trial is currently enrolling to evaluate the safety and efficacy of saracatinib for the treatment of IPF (identifier: NCT04598919).

Connective tissue growth factor (CTGF) is a pro-fibrotic matricellular protein that has been identified as a drug target for IPF. Plasma and lung tissue from patients with IPF have increased levels of CTGF, which directs fibroblasts, epithelial cells, and alveolar macrophages to promote fibrogenesis. 80 81 CTGF induces fibroblast proliferation and differentiation into myofibroblasts. 82 Both fibroblast-specific CTGF-deletion and inhibition using an anti-CTGF monoclonal antibody inhibited the development of bleomycin-induced fibrosis in mice. 83 84 Targeted CTGF inhibition as a treatment modality for IPF is being approached through the development of novel monoclonal antibodies. Pamrevlumab exhibited efficacy and safety in treating IPF in the phase II PRAISE trial. 85 Unfortunately, the phase III ZEPHYRUS-1 trial studying pamrevlumab did not meet its primary endpoint of change in FVC at 48 weeks (−330 mL in the placebo group and −260 mL in the pamrevlumab group; *p* = 0.29). 86 There was no significant difference in adverse events. Another monoclonal antibody targeting CTGF, SHR-1906, demonstrated safety in a phase I trial demonstrated safety, and a phase II trial of the agent is currently underway (identifier: NCT05722964). 87

Interleukin signaling has been another therapeutic target in IPF. IL-1β, IL-2, IL-6, IL-8, IL-10, IL-11, IL-12, IL17-A, and IL-33 are all increased in the blood or BAL of IPF patients compared to healthy controls, implicating a role for inflammation in the fibrotic lung. 88 89 These cytokines are largely pro-inflammatory, though some have pro-fibrotic activity as well, and facilitate immune cell recruitment, ECM production, and fibrosis. 88 90 Despite mouse models demonstrating a robust inflammatory response to bleomycin preceding fibrosis, targeting an inflammatory component of IPF has not proven beneficial, and may even worsen mortality. The PANTHER-IPF study, while limited by its multi-arm protocol and early termination, demonstrated that use of N-acetylcysteine (NAC) combined with the immunomodulatory agents prednisone and azathioprine was associated with an increased risk of death or hospitalization. 91 Additionally, inhibition of TNF-α with etanercept was shown to be an ineffective treatment modality for IPF. 92 Therefore, the use of anti-inflammatory maintenance therapy for IPF appears to be an ineffective strategy. IL-11 and members of the IL-6 family continue to hold promise as therapeutic targets in part due to their pro-fibrotic actions. IL-11 is increased in the lungs of IPF patients and positively correlates with disease severity. 89 In response to IL-11 stimulation, fibroblasts differentiate into myofibroblasts and produce collagen. Exogenous IL-11 administration and fibroblast-specific overexpression of IL-11 in vivo promoted the accumulation of collagen in the lungs of mice. 89 After bleomycin, deletion of an IL-11 receptor subunit inhibited the development of fibrosis in mice. 89 A neutralizing antibody to IL-11 receptor, LASN01, has been developed and has completed a phase I/IIa clinical trial with results pending formal publication (identifier: NCT05331300). Oncostatin M (OSM), a member of the IL-6 family of cytokines, is elevated in bronchoalveolar lavage (BAL) fluid of IPF patients. 93 OSM is not only pro-inflammatory, but also modulates ECM production, drives fibroblast proliferation, and inhibits fibroblast apoptosis. 93 94 95 Vixarelimab is a monoclonal antibody against the OSM receptor OSMRß and is being studied in a phase IIa trial in IPF (identifier: NCT05785624).

## Cyclic AMP, Cyclic GMP, Endothelin-1, and Thromboxane

Preclinical and clinical studies in IPF have also evaluated drugs classically used for pulmonary hypertension (PH) due to overlapping anti-fibrotic effects. Cyclic adenosine monophosphate (cAMP) is produced by adenylyl cyclase and activates cAMP-dependent protein kinase A (PKA) and exchange protein activated by cAMP, leading to downstream anti-inflammatory and anti-fibrotic effects in the lung. 96 97 98 99 100 Elevated cAMP levels inhibit TGF-β and Smad signaling, an effect reversed by PKA inhibition, suggesting cAMP and PKA play a critical role in modulating TGF-β signaling. 101 Prostacyclin and its analogues, including treprostinil, activate adenylyl cyclase to produce cAMP. 102 103 104 Treprostinil inhibits TGF-β secretion, fibroblast proliferation, myofibroblast differentiation, and collagen deposition and has been shown to inhibit the development of bleomycin-induced fibrosis in mice. 105 106 In the INCREASE trial, which evaluated inhaled treprostinil for treatment of ILD-associated PH, subgroup analyses showed a non-significant trend toward not only stable, but improved FVC in ILD patients, 28% of whom had IPF. 107 This formed the basis for the TETON trials, two phase III RCTs evaluating the efficacy of inhaled treprostinil in treating IPF, which are currently underway (identifiers: NCT04708782 and NCT05255991). 108

Phosphodiesterases (PDEs) are a family of enzymes that hydrolyze cAMP, cyclic guanosine monophosphate (cGMP), or both cAMP and cGMP. Given the crucial role of cAMP in pulmonary fibrosis, attention has turned to PDE4, a cAMP-specific PDE that increases intracellular cAMP. Nonspecific PDE4 inhibitors have been shown to inhibit development and therapeutically reduce fibrosis in multiple preclinical models of pulmonary fibrosis, including bleomycin. 109 110 111 However, the nonspecific PDE4 inhibitors' clinical utility has been hampered by adverse side effects, namely headaches, gastrointestinal, and psychiatric side effects. 112 Targeted PDE4B inhibition offers potent anti-fibrotic effects with a better side effect profile. Specifically, PDE4B inhibition with nerandomilast therapeutically reduced fibrosis in both bleomycin and silica models of pulmonary fibrosis in mice. 98 A phase II RCT of nerandomilast in IPF showed stabilization of FVC at 12 weeks either alone (−81.7 mL in the placebo group and +5.7 mL in the nerandomilast group) or in combination therapy with background anti-fibrotics (−59.2 mL in the placebo group and +2.7 mL in the nerandomilast group). 113 Diarrhea was the most common side effect. Among patients taking background anti-fibrotics, diarrhea occurred in 32% of placebo patients and 37% of nerandomilast patients, and in the absence of background therapy, diarrhea occurred in 16% of placebo patients and 27% of nerandomilast patients. The FIBRONEER trials, two phase III RCTs studying nerandomilast in IPF, have been completed, and while data has yet to be published, press releases report similar clinical benefit as its phase II counterpart. 114

cGMP is produced by guanylyl cyclase in response to nitric oxide and activates cGMP-dependent protein kinase G to exert anti-fibrotic effects. 115 PDE5 inhibitors like tadalafil and sildenafil are cGMP-specific and are clinically used to treat PH through their effect on vasodilation. Increased cGMP levels, driven by guanylyl cyclase stimulation, inhibit TGF-β-dependent production of collagen in human dermal fibroblasts. 115 116 Preclinical studies show that sildenafil inhibits fibrosis development in mice after bleomycin, with improved nitric oxide synthase coupling and a reduction in reactive oxidative species (ROS). 117 Clinical trials with sildenafil have proven less promising. STEP-IPF evaluated sildenafil for the treatment of IPF and found no significant improvement in their primary outcome of 6MWT improvement. They did identify minor improvements in DL _CO_ and quality of life. 118 INSTAGE sought to re-analyze sildenafil in a sicker patient cohort (DL _CO_ < 35% predicted) and in combination with nintedanib. Using the St. George Respiratory Questionnaire, no improvement in symptomatology was noted compared to nintedanib alone, but this may be limited by patients having more advanced disease at the outset of the study. 119

Like prostacyclins and PDE5 inhibitors, endothelin receptor antagonists (ERA) are a class of medications approved for the treatment of PH that have also been repurposed for study in pulmonary fibrosis. There are two G-protein-coupled receptor endothelin receptor subtypes found in humans that activate a variety of different downstream pathways, including PKC, AKT, and β-arrestin, and upon activation, can also stimulate IL-11 release through a MAPK-dependent pathway. 120 121 Endothelin-1 (ET-1) and its precursors are expressed by airway and alveolar epithelial cells, as well as inflammatory cells and alveolar macrophages. 122 123 ET-1 levels are increased in the serum of IPF patients. 124 ET-1 is known to promote fibroblast chemotaxis, proliferation, and protection against apoptosis while also stimulating ECM deposition. 125 126 127 128 Overexpression of ET-1 drives inflammation and fibrosis in mice, but endothelin antagonism with ERAs has shown mixed responses to bleomycin injury in rat models. 129 130 131 Nonetheless, ERAs advanced to clinical trials in IPF. The BUILD-1 trial evaluating bosentan in IPF patients did not demonstrate improvements in the primary endpoint of 6MWT distance at 12 months, but did show a non-significant trend to reduced mortality and disease progression. 132 Subgroup analysis demonstrated a significant mortality benefit in those with surgical biopsy-diagnosed IPF, leading to the BUILD-3 study. In BUILD-3, patients with biopsy-proven IPF were randomized to bosentan therapy or placebo, and no significant difference in the rate of disease progression was identified. 133 ARTEMIS-IPF evaluated ambrisentan therapy but was terminated early due to ambrisentan treatment being associated with increased risk of meeting the primary endpoint of progression as defined by time to death, respiratory hospitalization, or decrease in lung function. 134 Macitentan was studied in the MUSIC trial, where no differences were observed between the treatment and placebo groups. 135 Combined, these trials ended enthusiasm for ERAs as a therapeutic agent for IPF.

Thromboxanes are bioactive metabolites of arachidonic acid, like prostacyclins, and similarly have been studied in IPF. Increases in thromboxane relative to prostacyclin have been documented in IPF-fibroblasts in ex vivo analyses. 136 The thromboxane receptor TBXA2R has increased expression in the lungs of IPF patients, especially fibroblasts, endothelial cells, and smooth muscle cells. 137 Genetic deletion of TBXA2R in mice inhibited the development of fibrosis after bleomycin, and inhibition of TBXA2R with the small molecule inhibitor ifetroban improved fibrosis in multiple mouse models (bleomycin, genetic, and radiation). 137 Ifetroban is now under investigation in a phase II RCT for the treatment of IPF (identifier: NCT05571059). While trials for sildenafil and ERAs have not demonstrated significant clinical efficacy, targeting thromboxane and cAMP signaling remains under investigation and has shown early therapeutic potential in IPF.

## Autotaxin, Lysophosphatidic Acid, and Lysophosphatidic Acid Receptor

The autotaxin (ATX)-lysophosphatidic acid (LPA)-LPA receptor (LPAR) axis has shown both promising and dangerous potential for treating IPF. Autotaxin is an excreted enzyme that converts lysophosphatidylcholine to LPA and serves as an LPA chaperone to its receptor LPAR. 138 139 140 141 There are many isoforms of LPA with different saturations and lengths of fatty acid chains, and six isoforms of the G-coupled protein receptors LPAR (LPAR1-6). 139 LPARs have varying affinities for species of LPA, with LPAR1 being the primary binding partner of LPA. 139 ATX-LPA-LPAR signaling can be both pro-inflammatory and pro-fibrotic. In pulmonary fibrosis, this axis participates in crucial events including epithelial cell apoptosis, TGF-b activation, and fibroblast recruitment, activation, and survival through several signaling pathways including the Ras-Raf-MEK-ERK, PI3K, Rho/ROCK, Rac, and phospholipase C pathways. 140 141 142 143 144 145 146 Increased staining for ATX has been demonstrated within the hyperplastic bronchiolar epithelium, alveolar epithelium adjacent to ﬁbroblastic foci, and fibroblasts and alveolar macrophages within the fibrotic interstitium of IPF lungs. 147 Elevated levels of LPA have been measured in the BAL of patients with IPF, and higher levels are associated with worse DL _CO_ and radiographic fibrosis. 144 148 Development of bleomycin-induced fibrosis was inhibited with genetic deletion of ATX in macrophages and bronchial epithelial cells, and after therapeutic inhibition of ATX with ziritaxestat and GWJ-A-23. 147 149 Additionally, ziritaxestat decreased established radiation-induced fibrosis in mice. 140 Similarly, genetic knockout of LPAR1 and LPAR1 inhibition with AM966 both inhibited development of bleomycin-induced fibrosis. 144 150 An LPAR 1/3 inhibitor was also shown to reduce the development of radiation fibrosis in mice. 151 While phase II clinical trials for ATX inhibitors, cudexestat (RESPIRARE trial) and BBT-877 are yet to be published, ziritaxestat slowed FVC decline at 12 weeks in a phase II RCT. 152 However, two phase III RCTs of ziritaxestat in IPF (ISABELA 1 and 2) were terminated early due to a signal for increased mortality and lack of efficacy. 153 Treatment groups included placebo and ziritaxestat at both lower (200 mg daily) and higher (600 mg daily) doses for at least 52 weeks, and allowed for background therapy with nintedanib or pirfenidone. No difference was observed in the primary endpoint of annual rate of FVC decline (−145 mL with placebo, −174 with low dose, and −125 mL with high dose in ISABELA 1; −177 mL with placebo, −175 mL with low dose, and −174 mL with high dose in ISABELA 2). Secondary endpoints revealed no benefit, and time to first respiratory-related hospitalization was worse in the ziritaxestat group. Importantly, ziritaxestat has a drug-drug interaction with nintedanib, which may have contributed to adverse events. ATX levels were increased in the plasma of treated patients in the ISABELA 1 and 2 trials, suggesting a regulatory feedback loop that could explain the lack of efficacy and adverse effects. 154 Despite the signal for harm with ziritaxestat, LPAR1 inhibition continues to be studied. LPAR1 antagonists in clinical trials include fipaxalparant and admilparant. BMS 986020 demonstrated promising efficacy in a phase II RCT; however, it was terminated early due to inciting hepatobiliary disease. 155 In a phase II RCT of admilparant, patients were randomized to placebo, admilparant 30 mg or 60 mg twice daily, and permitted to continue background nintedanib or pirfenidone. The mean rate of decline in FVC was −2.7, −2.8, and −1.2% in the placebo, 30 and 60 mg groups, respectively. 156 Serious adverse events occurred in 17% of the placebo group, and 11% of both 30 and 60 mg groups. There was an increased frequency of transient day 1 postdose blood pressure reduction with admilparant, but no increased frequency of diarrhea. A more pronounced benefit was seen in a progressive pulmonary fibrosis (PPF) cohort, with the decline in FVC of −4.3, −2.7, and −1.1% in the placebo, 30 mg, and 60 mg groups, respectively. This led to a phase III RCT with admilparant which is underway (identifier: NCT06003426). Despite the failure of ziritaxestat, targeting the ATX-LPA-LPAR axis remains a promising therapeutic strategy.

## Extracellular Matrix and Integrins

Excessive deposition and accumulation of ECM is a hallmark feature of IPF. A positive feedback loop exists whereby increased deposition of ECM increases lung stiffness and promotes pro-fibrotic cellular responses via mechanotransduction. 157 Therefore, targeting critical ECM components to break this positive feedback loop holds potential as a therapeutic avenue in IPF.

Integrins are heterodimeric, transmembrane glycoprotein receptors that consist of an α-subunit and a β-subunit. 158 They are cell adhesion signaling proteins, with the unique ability to transduce signals bidirectionally from the intracellular environment to elicit changes in the extracellular environment, and vice versa, allowing cells to sense and respond to ECM mechanical changes. 158 Clinically, integrin inhibitors are commonplace, like abciximab (Reopro), eptifibatide (Integrilin), or tirofiban (Aggrastat), which inhibit αIIbβ3 on platelets and are used during percutaneous coronary interventions, or the pan-α4 inhibitor natalizumab (Tysabri), which is used for multiple sclerosis treatment. 158 159 160 Integrins play a pivotal function in IPF, in large part due to their role in TGF-β activation. When inactive, TGF-β is bound to latency-associated peptide and tethered to the ECM. 158 161 162 163 Multiple integrins, including αvβ6 on epithelial cells and αvβ1 on fibroblasts, release TGF-β from its inactive state. 158 161 162 αvβ6 protein expression is increased in IPF tissue compared to healthy controls, localizes to epithelial cells overlying fibrotic areas and to areas with active inflammation, and has been associated with increased mortality. 164 165 Targeting αvβ6 has been a promising option to treat pulmonary fibrosis. Genetic knockout of β6 (preventing formation of αvβ6) protected mice from bleomycin-induced fibrosis, and therapeutic inhibition of αvβ6 after bleomycin inhibits development, therapeutically reduces, and accelerates resolution of fibrosis in mice. 161 164 166 Similarly, inhibition of αvβ1 therapeutically reduced fibrosis in mice after bleomycin. 162 A monoclonal antibody against αvβ6 (BG00011) was evaluated in a phase IIb RCT in IPF, but was terminated early due to lack of benefit and increased adverse events, including increased progression, exacerbations, and death in the treatment group compared to placebo. 167 A question remains as to whether monoclonal antibodies can permeate into the dense fibrotic regions of IPF lungs, and if small-molecule inhibitors will therefore prove superior. Bexotegrast, a dual αvβ1 and αvβ6 small molecule inhibitor, targets both epithelial (αvβ6) and fibroblast (αvβ1) integrins mediating TGF-β activation. INTEGRIS-IPF was a phase IIa RCT of patients with IPF receiving once-daily placebo or bexotegrast (40, 80, 160, or 320 mg groups) with or without background nintedanib or pirfenidone over at least 12 weeks. 168 The trial found a similar incidence of adverse events, with the most common side effect being diarrhea, which was primarily seen in patients co-treated with nintedanib. Grade 3 or greater adverse events occurred in 6.5% of placebo-treated patients and 6.7% of bexotegrast-treated patients. FVC decline, CT quantification of fibrosis, and biomarkers all demonstrated a benefit in the bexotegrast groups over the 12-week trial period. A phase IIb/III trial (BEACON-IPF) of bexotegrast in IPF recently stopped enrollment and dosing after recommendations from an independent Data Safety Monitoring Board, but the reasons have yet to be published. 169

Excessive collagen synthesis, accumulation, maturation, and impaired degradation all contribute to the progression of fibrosis, making targeting collagen an appealing therapeutic strategy. Type 1 collagen is made of two α _1_ chains (encoded by COL1A1) and one α _2_ chain (encoded by COL1A2). Prepro-polypeptide chains travel to the endoplasmic reticulum (ER) where they undergo posttranslational modification and assembly of a triple helix, then are transported to the Golgi apparatus in the form of pro-collagen for further processing and assembly into secretory vesicles, are secreted, and subsequently undergo extracellular modifications, including collagen crosslinking. 170 Several steps in the collagen biosynthesis and posttranslational modification are under investigation as therapeutic targets for IPF.

Prolyl-tRNA synthetase (PRS) conjugates proline to tRNA during collagen translation. 171 DWN12088, a PRS inhibitor, therapeutically reduced bleomycin-induced fibrosis in mice, which has led to a current phase II RCT to evaluate its safety and efficacy in IPF (identifier: NCT05389215). 171

Heat shock protein 47 (HSP47) is a collagen-specific chaperone that is essential for the correct folding of pro-collagen fibrils in the ER. 172 HSP47 also plays a role in collagen turnover through collagenase, MMPs, and integrin signaling. 172 A phase II RCT with siRNA targeted HSP47 gene silencing (ND-L02-s0201) did not demonstrate efficacy in patients with IPF (identifier: NCT03538301).

Lysyl oxidase-like 2 (LOXL2) catalyzes collagen crosslinking and has increased expression in fibrotic areas of IPF lung tissue. 173 Preclinical studies showed that inhibition of LOXL2 inhibited development and therapeutically reduced fibrosis after bleomycin treatment in mice. 173 However, a phase II RCT of a LOXL2 inhibiting monoclonal antibody, simtuzumab, in IPF; however, was terminated early for futility. 174 Transglutaminase-2 (also known as tissue transglutaminase), which crosslinks ECM components, is currently being investigated in a phase II RCT with the transglutaminase-2 inhibitor, GSK3915393, in IPF (identifier: NCT06317285). 175

## Senescence

In its basic definition, senescence refers to the arrest of cellular growth and is a key pathologic feature of IPF. In IPF, senescent AEC2s have reduced regenerative capacity, and senescent fibroblasts develop resistance to apoptosis. 176 Senescent cells also secrete pro-fibrotic and pro-inflammatory cytokines, chemokines, growth factors, and proteases, referred to as a senescence-associated secretory phenotype. 176 Senescent fibroblast resistance to apoptosis is in part due to increased expression of anti-apoptotic BCL-2 family members, including BCL-2 and BCL-XL, which have been shown to increase in response to stiffness of fibrotic lungs. 177 BH3 mimetics are drugs that were initially developed as anti-cancer agents, inhibit anti-apoptotic BCL-2 family proteins, and include the BCL-2 specific inhibitor venetoclax and the BCL-2, BCL-XL, and BCL-W inhibitor navitoclax. Navitoclax has been shown to reduce the presence of senescent cells and decrease fibrosis after radiation-induced fibrosis in mice. 178 Furthermore, navitoclax and venetoclax have been shown to inhibit development and therapeutically reduce bleomycin-induced fibrosis in mice. 179 180 181 We have recently shown that using a repetitive dosing regimen of bleomycin, which induces PPF, navitoclax stabilized progressive fibrosis by targeting pro-fibrotic fibroblasts for apoptosis. 182 While navitoclax remains a study drug in cancer clinical trials in large part due to its side effect of thrombocytopenia, venetoclax is FDA approved for chronic lymphocytic leukemia and acute myeloid leukemia. A small phase 1 clinical trial evaluating its safety and efficacy in IPF (identifier: NCT05976217) was recently completed, with the results pending publication. The senolytic combination of dasatinib and quercitin has also shown promise as a potential treatment for IPF. Dasatinib is an inhibitor of multiple tyrosine kinases, and quercitin is a natural flavonoid. Together, they have been shown to target senescent cells and inhibit the development of bleomycin fibrosis in mice. 176 A small single-center study evaluated the feasibility of dasatinib plus quercitin in IPF. 183 It demonstrated drug tolerability, and while it did not find changes in disease severity after treatment, it was underpowered. Due to the crucial mechanistic role of senescent cells in IPF, senolytics hold promise as therapeutic agents for IPF.

## Reactive Oxygen Species, Unfolded Protein Response, Endoplasmic Reticulum Stress, and Metabolic Stress

Metabolic stress, whether ROS or ER stress, can drive epithelial injury and promote fibrotic responses. ROS are known to stimulate a pro-fibrotic environment through increased release of TGF-β, which in turn stimulates further ROS production. 184 185 186 IPF fibroblasts express NADPH oxidase isoform 4 (Nox4) in response to TGF-β, which drives the formation of ROS and facilitates a positive feedback loop promoting fibrogenesis. 187 188 189 siRNA knockdown of Nox4 inhibited the development of bleomycin-induced fibrosis in mice. 187 Similarly, Nox1 and Nox4 inhibition with setanaxib (GKT137831) hastened the resolution of bleomycin fibrosis in mice while also decreasing myofibroblast accumulation and improving mortality. 190 Notably, these studies were performed in aged mice, thus more closely mimicking the advanced age of IPF patients. A phase IIb trial of setanaxib was recently completed in IPF patients, and results are pending publication (identifier: NCT03865927).

The ER is a vital cellular organelle involved in protein translation and trafficking. ER stress occurs when these processes are disrupted, particularly in the setting of aberrant protein folding, leading to an unfolded protein response (UPR). UPR was first identified as a pathophysiologic mechanism for fibrosis when mutated surfactant protein C led to misfolding in the ER of alveolar epithelial cells, eventually leading to pulmonary fibrosis. 191 192 193 194 195 UPR promotes fibrosis by inducing fibroblasts to myofibroblast differentiation and AEC2 apoptosis. 196 197 198 IRE1a is an ER sensor that drives UPR. Upon activation, IRE1a auto-phosphorylates, oligomerizes, and cleaves XBP1 mRNA, allowing for translation of XBP1, which drives downstream expression of ER stress genes. 199 In response to lung injury and UPR, IRE1a enhances TGF-β signaling. IRE1a deletion and therapeutic antagonism of IRE1a inhibit the development of bleomycin-induced fibrosis, and even promote type 2 alveolar epithelial cell growth and tissue repair. 200 201 ORIN1001, which inhibits the RNase activity of IRE1a, is under investigation in a phase Ib clinical trial in IPF patients that is currently suspended (identifier: NCT04643769).

## Stem Cells

While preclinical and clinical studies to date have largely focused on halting or slowing fibrosis, stem cells offer a novel method to regenerate damaged tissue. Initial interest in the field focused on mesenchymal stromal cells (MSCs). Animal studies showed that the introduction of MSCs inhibited the development, reduced, and hastened the resolution of bleomycin-induced fibrosis. 202 203 MSCs function by promoting anti-inflammatory and anti-fibrotic signaling, reducing metabolic stress, and, to a lesser extent, engrafting into the lung. 204 205 206 207 208 209 210 While the phase I AETHER study of adipose-derived MSCs documented safety, a randomized, open-label phase Ib/IIa trial showed improvement in 6MWT distance, DL _CO_ , and FVC using placental-derived MSCs. 211 212 Additional phase I trials are ongoing with umbilical cord-derived MSCs, including a phase Ib/IIa trial (identifier: NCT05468502).

Lung-derived spheroid stem cells (LSC) are another option being explored for the treatment of IPF. LSCs can be obtained autologously from lung tissue, generally obtained through biopsy. 213 The tissue is applied to an adherent surface, and migrating cells, termed explant-derived cells, are then collected and cultured in suspension. While in suspension, these cells form spheroids, which are associated with increased expression of progenitor markers. 214 An allogeneic rat model of LSC injection demonstrated reduced inflammation and inhibition of fibrosis development after bleomycin compared to controls, without generating a significant alloimmune response. 214 A phase I RCT is currently recruiting patients to establish the safety of LSCs in humans (identifier: NCT04262167). Stem cells provide an exciting new prospect for IPF therapeutics, but given limited phase II trials and no phase III trials, their therapeutic potential remains in the early stages.

## Angiotensin

While the pulmonary renin-angiotensin system (PRAS) has long been an area of study in lung injury and fibrosis, it was brought to the forefront when the COVID-19 pandemic highlighted the significance of bronchoalveolar epithelial expression of angiotensin-converting enzyme 2 (ACE2). ACE2 was implicated in the pathogenesis of disease and in postinfectious fibrotic sequelae of COVID-19. 215 216 217 ACE2 is a membrane-bound protein that converts angiotensin I and angiotensin II to Ang-(1-9) and Ang-(1-7), respectively. 218 ACE2 is necessary to maintain homeostasis of PRAS. Decreasing ACE2 activity can lead to elevated angiotensin II levels in the lung, which, upon binding to the angiotensin type 1 receptor (AT _1_ ), leads to alveolar epithelial cell apoptosis. 219 220 ACE2 has decreased transcript expression and enzymatic activity in biopsy specimens from IPF patients. 221 Apoptotic alveolar epithelial cells in IPF patients release angiotensinogen, which will convert to angiotensin II and drive downstream epithelial apoptosis and promote collagen production by fibroblasts. 222 223 224 Exogenous ACE2 co-administered with bleomycin protected against epithelial cell injury, decreased TGF-B expression, decreased a-SMA expression, and inhibited the development of fibrosis in mice. 225 ACE2 null mice experienced exaggerated fibrosis in response to bleomycin. 226 Notably, the ACE2-Ang-(1-7)-Mas pathway leads to increased expression of angiotensin type 2 receptor (AT _2_ ), which, in opposition to AT _1_ , drives anti-fibrotic responses and is protective against lung injury. 227 228 229 230 231 Indeed, buloxibutid, an AT _2_ agonist, was shown to inhibit bleomycin-induced fibrosis. 232 An open-label phase II trial of buloxibutid for treatment in IPF has been completed (identifier: NCT04533022), and results have not been formally published. The ASPIRE trial is a phase II RCT studying Buloxibutid treatment in IPF, is currently recruiting (identifier: NCT06588686). These studies will provide unique clinical insight into the supportive role ACE2 and AT _2_ play in maintaining alveolar integrity to treat IPF.

## Pentraxin

Pentraxin-2, also known as serum amyloid P, signals through Fcγ and exerts anti-fibrotic action through modulating pro-fibrotic monocyte-derived macrophages and fibrocytes. 233 234 IPF patients have lower plasma pentraxin-2, and lower levels correlate with disease severity. 235 Genetic loss of pentraxin-2 in mice results in an exaggerated fibrotic response after bleomycin, while pentraxin-2 treatment inhibits bleomycin and TGF-b-induced fibrosis development. 233 234 235 236 A phase II RCT evaluated recombinant human pentraxin-2 in IPF and found that after 28 weeks, the decline in FVC was −4.8 and −2.5% in the placebo and pentraxin-2 groups, respectively. 237 This led to the phase III RCT STARSCAPE evaluating pentraxin-2 in IPF. 238 However, STARSCAPE was terminated early for futility. At 52 weeks, the decline of FVC in placebo and pentraxin 2 was −215 and −236 mL, respectively.

## Discussion

An abundance of preclinical studies have revealed many promising therapeutic targets to help people suffering from IPF. Unfortunately, very few drugs make it to phase II or III clinical trials, and only three drugs have shown efficacy in treating IPF in phase III trials (nintedanib, pirfenidone, and nerandomilast). A major obstacle is that many of these drug therapies are “lost in translation,” and successful in vivo studies are not consistently translating to effective bedside treatments. One major reason behind this failure may be the systems used to evaluate for preclinical efficacy. The translational science toolkit includes in vitro cell culture systems, ex vivo precision-cut lung slices from healthy and diseased animals and humans, in silico modeling or simulation, and in vivo animal models, most often mice. A critical component of preclinical projects is often in vivo mouse work, demonstrating efficacy by improving fibrosis. The most widely used model is single-dose intratracheal bleomycin in young mice (8–12 weeks), which undergoes spontaneous resolution had has limited pathologic overlap with IPF. Risk factors for IPF include genetic predisposition, age, smoking, and other environmental exposures. This starkly contrasts with studies using genetically identical young mice, living in a pathogen-free environment. Additionally, mice lack respiratory bronchioles and spend their lives in the prone position. Mice aged 8 to 12 weeks roughly translate to 20-year-old humans, while mice aged 18 to 24 months roughly translate to 56 to 69-year-old humans, the age at which patients are typically diagnosed with IPF. 21 Therefore, studying “middle-aged” or older mice may offer a better model of IPF than young mice, especially when studying treatment options. Additionally, how investigators utilize this model to test drugs is often inappropriate. Intratracheal bleomycin induces an early, robust inflammatory phase, followed by a fibroproliferative and established fibrotic phases, with subsequent spontaneous resolution. 21 Between 2008 and 2019, single-dose bleomycin was used to investigate 726 potential therapies. 23 The majority (61%) were tested as a preventative of fibrosis, as they were given prior to the establishment of fibrosis. Twenty-three percent were assessed for “therapeutic” effect as treatment was given during the fibroproliferative phase, while 14.5% were tested as both preventative and therapeutic. 23 Prior to 2008, less than 5% of bleomycin drug studies were “therapeutic.” 23 We believe that utilizing more clinically relevant models of progressive fibrosis, like repetitive bleomycin, aged mice, or mice with relevant genetic mutations like MUC5B or TERT mutations, represents a significant paradigm shift that preclinical science must consider adopting, despite the protracted nature of such studies. In the future, utilizing other animal models, like ferrets, which have respiratory bronchioles and develop persistent fibrosis after bleomycin, or West Highland White Terriers, who develop spontaneous PPF, may become more prevalent. 239 240 Nonetheless, in vivo mouse studies, in particular single-dose bleomycin, have been integral to our understanding of the pathobiology of pulmonary fibrosis.

Selection of primary endpoints also bears consideration when interpreting clinical trials. IPF trials have typically used FVC as the primary endpoint to determine the efficacy of the intervention, comparing the amount of decline at 6 or 12 months to determine progression. The long duration of these studies to identify evidence of progression adds to the high expense of clinical trials in IPF. Recently, it has been proposed that 3-month changes may be predictive of progression and could be used as an endpoint, thereby shortening study duration, which could improve cost and potentially lead to more therapeutic agents being tested. 241 Furthermore, while a relatively easy measure to obtain, the utility of FVC as the primary endpoint has been called into question. As an effort-dependent measurement, there can be variability between measurements. Also, it may not account for all important patient-reported outcomes such as progressive symptoms of dyspnea, cough, or decline in physical function. 15 Moving forward, composite endpoints including FVC, patient-reported outcomes, and potentially novel biomarkers could aid in future clinical trial design.

While several promising therapeutic targets offer potential treatments for IPF, there remain several untapped resources that could prove helpful in improving the lives of people with IPF. First, utilizing new technologies such as artificial intelligence (AI) may be one way to expedite the translation of basic science work to clinical trials. For example, ISM001-005, a first-in-class small molecule inhibitor of TRAF2 and NCK-interacting kinase (TNIK), was designed using generative AI. It has had positive results from its Phase IIa trial per the company's website, though a formal publication is still pending. 242 Second, precision medicine offers new opportunities for people living with IPF. For example, a retrospective analysis of the PANTHER trial, INSPIRE trial, and University of Chicago IPF cohorts found that NAC treatment was beneficial in IPF patients carrying the TOLLIP rs3750920 TT genotype and potentially harmful in those with the rs3750920 CC genotype. 243 Third, comorbidities need to be a focus of future research, as there is a clear interplay between IPF and comorbidities like GERD, PH, and obstructive sleep apnea. For example, inhaled treprostinil has been shown to improve 6MWT in ILD patients with PH. 107 Rather than the “one size fits all” approach used in IPF treatment, delineating effects with certain genotypic or phenotypic variations of IPF could prove exceedingly helpful. Fourth, early diagnosis of people with interstitial lung abnormalities and delineation of which patients will progress is critical and has the potential to allow for early intervention that could halt progressive fibrosis in its tracks. Finally, combination therapy will likely become the standard of care as more therapies become available. Combination therapy is a widely used treatment approach for diseases including COPD, asthma, hypertension, diabetes, HIV, and cancer. Clearly, multiple pathways are involved in the pathogenesis of IPF, as described above. Targeting multiple pathways allows for therapeutic synergy, additive effects, and dose reductions to avoid adverse effects.

The next several years will be an exciting time for IPF drug development. Substantial preclinical data have provided numerous promising candidate pathways, and several encouraging clinical trials are underway. The FIBRONEER trial of nerandomilast in IPF demonstrated efficacy of PDE4B inhibition, becoming the first positive phase III RCT in IPF in a decade. 114 Currently, two other medications (treprostinil and admilparant) are under investigation in phase III RCTs. As detailed above, there are several other targets with strong preclinical rationale and encouraging phase II RCT results. We anticipate several more options to become available for patients within the next decade to help relieve the suffering, economic burden, and early mortality associated with IPF.
